# Genetic Evidence of Contemporary Dispersal of the Intermediate Snail Host of *Schistosoma japonicum*: Movement of an NTD Host Is Facilitated by Land Use and Landscape Connectivity

**DOI:** 10.1371/journal.pntd.0005151

**Published:** 2016-12-15

**Authors:** Jennifer R. Head, Howard Chang, Qunna Li, Christopher M. Hoover, Thomas Wilke, Catharina Clewing, Elizabeth J. Carlton, Song Liang, Ding Lu, Bo Zhong, Justin V. Remais

**Affiliations:** 1 Department of Environmental Health, Rollins School of Public Health, Emory University, Atlanta, Georgia, United States of America; 2 Department of Biostatistics and Bioinformatics, Rollins School of Public Health, Emory University, Atlanta, Georgia, United States of America; 3 Environmental Health Sciences, School of Public Health, University of California, Berkeley, California, United States of America; 4 Department of Animal Ecology and Systematics, Justus Liebig University, Giessen, Germany; 5 Department of Environmental and Occupational Health, Colorado School of Public Health, University of Colorado, Anschutz, Aurora, Colorado, United States of America; 6 Department of Environmental & Global Health, College of Public Health and Health Professions, University of Florida, Gainesville, Florida, United States of America; 7 Institute of Parasitic Diseases, Sichuan Center for Disease Control and Prevention, Chengdu, China; FIOCRUZ - Minas, BRAZIL

## Abstract

**Background:**

While the dispersal of hosts and vectors—through active or passive movement—is known to facilitate the spread and re-emergence of certain infectious diseases, little is known about the movement ecology of *Oncomelania* spp., intermediate snail host of the parasite *Schistosoma japonicum*, and its consequences for the spread of schistosomiasis in East and Southeast Asia. In China, despite intense control programs aimed at preventing schistosomiasis transmission, there is evidence in recent years of re-emergence and persistence of infection in some areas, as well as an increase in the spatial extent of the snail host. A quantitative understanding of the dispersal characteristics of the intermediate host can provide new insights into the spatial dynamics of transmission, and can assist public health officials in limiting the geographic spread of infection.

**Methodology/Principal findings:**

*Oncomelania hupensis robertsoni* snails (n = 833) were sampled from 29 sites in Sichuan, China, genotyped, and analyzed using Bayesian assignment to estimate the rate of recent snail migration across sites. Landscape connectivity between each site pair was estimated using the geographic distance distributions derived from nine environmental models: Euclidean, topography, incline, wetness, land use, watershed, stream use, streams and channels, and stream velocity. Among sites, 14.4% to 32.8% of sampled snails were identified as recent migrants, with 20 sites comprising >20% migrants. Migration rates were generally low between sites, but at 8 sites, over 10% of the overall host population originated from one proximal site. Greater landscape connectivity was significantly associated with increased odds of migration, with the minimum path distance (as opposed to median or first quartile) emerging as the strongest predictor across all environmental models. Models accounting for land use explained the largest proportion of the variance in migration rates between sites. A greater number of irrigation channels leading into a site was associated with an increase in the site’s propensity to both attract and retain snails.

**Conclusions/Significance:**

Our findings have important implications for controlling the geographic spread of schistosomiasis in China, through improved understanding of the dispersal capacity of the parasite’s intermediate host.

## Author Summary

In China, human schistosomiasis is caused by infection with the parasitic blood fluke *Schistosoma japonicum*, which requires snail hosts as a lifecycle intermediary. Snail control efforts have been a key component of China’s schistosomiasis control program, which has reduced human infections from 11 million in the 1950s to approximately 115,000 today. However, schistosomiasis has re-emerged in some areas, and the range of areas infested by or suitable for snail hosts is expanding. Understanding how the physical structure of the environment influences snail migration could aid in understanding how intermediate host mobility contributes to persistence and/or re-emergence of schistosomiasis. Within the 29 sites sampled for snail hosts, we estimated that between 14–33% of snails were recent migrants from another site, and above average inter-site migration rates occurred between sites separated by up to 44 km. Greater landscape connectivity was associated with increased recent migration rates. Connectivity models considering land use patterns explained the largest proportion of the variance in migration rates between sites. An increase in the number of irrigation channels leading into the site was associated with an increase in a site’s propensity to both attract migrant snails and retain snails. Our findings have important implications for understanding and responding to the geographic spread of schistosomiasis.

## Introduction

The current distribution, and potential future spatial spread, of disease carrying hosts and vectors are influenced by their dispersal capacities and the characteristics of the landscape they inhabit. Quantitative characterizations of host and vector dispersal have proven useful in the development of effective control strategies for a range of pathogens [1–3]. Traditional approaches to estimating host and vector migration rates have relied on studies that release and recapture individuals with physical or chemical marks, but these techniques are impractical for large populations that exchange small numbers of migrants because the number of recaptures is often too low to infer migration patterns [4, 5]. As an alternative, multilocus genotype data have been used to estimate genetic diversity, gene flow, and migration rates of populations in a wide variety of systems [6–9]. Recently, Hauswald et al. suggested that, given the high degree of genetic diversity within the species, microsatellite data could be used to characterize migration of *Oncomelania hupensis*, the intermediate host of *Schistosoma japonicum*, the parasite that causes human schistosomiasis in East and Southeast Asia [10].

Host and vector migration are influenced by the physical structure of the environment, which can be summarized through measures of landscape connectivity [11–13] that describe the degree to which landscape units facilitate or limit the dispersal of a target organism, accounting for both geographic distance and environmental features that aid or impede movement [14]. Landscape connectivity models have been used to characterize the determinants of dispersal for a range of species, particularly where Euclidean distance alone captures insufficient detail with respect to landscape structure [14–18]. In addition to considering the role of landscape heterogeneity between sites, some models consider specific properties of sites themselves that can enhance or diminish origin-to-destination flows, leading to superior estimates of the determinants of inter-site dispersal [19, 20]. The effect of such site-specific properties can be examined through random effect models that directly estimate the relative in-migrant pull (termed attractivity) or out-migrant push (the converse of which is termed retentivity) of a site [21].

Quantitative characterization in this manner of the migration of *Oncomelania* spp. in China would be especially valuable in the context of ongoing efforts to control and ultimately eliminate schistosomiasis in the country [10]. Control of *Oncomelania* spp. has been a key component of the national schistosomiasis control program [11, 22–26], which has reduced human infections with *Schistosoma japonicum* from roughly 11 million in the 1950s to approximately 115,000 in 2014 [27, 28] and the area of snail habitat in China’s 12 current or formerly endemic provinces by more than 70% over the same period [29, 30]. However, there is evidence in recent years of re-emergence and persistence of infection in some areas [30, 31], despite intense control programs including human treatment, improvements to sanitation infrastructure, and snail control [28]. In Sichuan Province, schistosomiasis was found to have re-emerged in 8 of 46 counties that had previously met the criteria for designation as having achieved transmission control [32]. Meanwhile, the detection of *Oncomelania* spp. populations in new areas suggests the range of the intermediate snail host is expanding within China [31], and rising temperatures due to global climate change may further this expansion [33–35]. Understanding the dispersal characteristics of this intermediate host can provide new insights into the spatial dynamics of transmission, and can assist public health officials in limiting the geographic spread of infection.

Relatively little is known regarding the movement ecology of *Oncomelania* spp. The objectives of this study are to characterize the range of distances traversed by recently migrating *O*. *hupensis robertsoni* snails (i.e., within the current and previous two generations) and determine the landscape and other geographic features that influence their dispersal. We use microsatellite markers of *O*. *hupensis robertsoni* “populations” to estimate inter-village, bi-directional migration between 29 study sites in Sichuan Province. Using random effects models and characteristics of the intervening landscape, we determine the environmental drivers of migration as well as features of the sites that influence their attractivity and retentivity with respect to snail migrants.

## Materials and Methods

### Study areas

**Fig 1:**
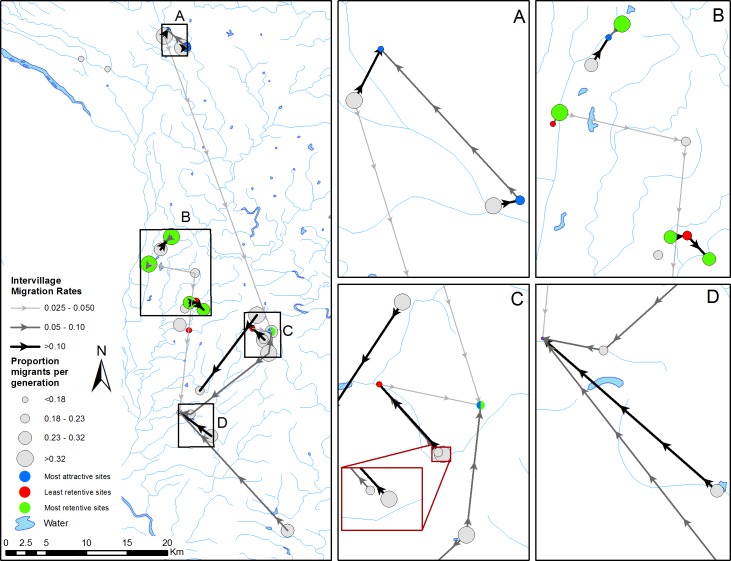
Map of study sites showing proportion of recent migrant individuals (denoted by symbol size) estimated by BayesAss at each site, propensity of the site to attract or retain migrants (denoted by symbol color; note two sites with split color symbology indicate simultaneous high attractivity and low/high retentivity), and inter-village migration rates (denoted by arrows). To improve clarity, inter-village migration rates less than 0.02 are not shown.

Within the eastern mountainous zone of Sichuan Province, 29 sites across 3 counties were selected for study on the basis of their recent history of re-emergence of *Schistosoma japonicum* in either snails or humans following previous transmission control [32]. Site boundaries were defined by the smallest community unit—the natural village or production group—referred to here as *villages* [36]. Shown in Fig 1, villages within this region lie along a network of rivers and streams that flow predominantly north to south, and the largest straight-line distance between sites is approximately 67 km. The average village in the region had approximately 80 people over 6 years of age (range 30-170 people), based on a census conducted in 2007 [36]. The region is characterized by mountainous terrain and intense agricultural cultivation, particularly rice and vegetable crops [15]. Agricultural practices rely on irrigation networks that may facilitate the dispersal of the intermediate host. Additionally, the common practice of applying human waste—in which parasitic eggs of infected hosts are shed—as crop fertilizer aides the transmission of schistosomiasis through the dispersal of eggs and associated larval stages.

### Landscape connectivity

Landscape connectivity between all study sites was determined by calculating the cumulative cost of passing through a rasterized (30m x 30m) depiction of the landscape lying between every pair of study sites, where the cost of passing through a grid cell reflects the habitat preferences of the intermediate host. Nine landscape connectivity models were developed using ArcGIS Model Builder and the cost distance toolset [37], automated in Python [38] and then used to estimate measures of connectivity between study sites by modifying the cost of specific landscape properties that facilitate or limit snail dispersal (Table 1). The landscape connectivity models included: Euclidean, topography, incline, wetness, land use, distance from watershed, stream use, streams and channels, and stream velocity. Euclidean distance measures the straight-line distance between two sites, ignoring landscape and topographic factors. Topography and incline models account for isotropic and anisotropic changes in elevation between sites, respectively. Other models considered the role of stream movement (stream only, stream velocity, streams and channels), soil wetness and water, and land use features in determining intermediate host dispersal (Table 1). Landsat and IKONOS imagery was mosaicked using ENVI (ITT Visual Information Solutions, Boulder, Colorado, USA) and land cover was classified using a maximum likelihood supervised classification approach with five classes—surface water, agriculture, forest, barren, and built, in order of least to greatest resistance to host movement—following previous methods [39, 40].

**Table 1:** Ecological properties considered in the development of connectivity models.

Model	Description	High Cost Features	Low Cost Features
**Euclidean**	Dispersal is limited by straight-line distance	-	-
**Topography**	Dispersal is limited by overland distance, accounting for elevation change	High elevations	Low elevations
**Incline**	Dispersal uphill is limited, downhill is facilitated	Uphill movement	Downhill movement
**Wetness**	Dispersal is facilitated by increased soil wetness	Dry soil	Wet soil
**Land use**	Dispersal is facilitated or limited by certain land cover classifications	Still water, barren and built land	Agricultural land and streams
**Watershed**	Dispersal is facilitated as distance from nearest stream cell decreases and is limited as distance from nearest stream cell increases	Areas far from stream cells	Areas close to stream cells
**Stream use**	Dispersal is facilitated along streams, and limited in the upstream direction	Land	Streams
**Streams and channels**	Dispersal is facilitated along streams and within the agricultural channel network, and limited in the upstream direction	Land	Streams and agricultural channels
**Stream velocity**	Dispersal is facilitated along streams, and limited in the upstream direction, proportional to flow velocity in the water channel	Land	Streams

The cost of every possible path between each pair of sites across the study region was calculated, yielding a distribution of costs as described elsewhere [15]. The minimum value of this distribution is generally taken as a summary measure of effective distance between sites, yet some have argued that such minima are insufficient to characterize landscape connectivity across complex landscapes [41–43]. In the current work, three summary measures of the distribution of path costs were examined: the minimum, the first quartile, and the median.

### Determination of village characteristics

Village characteristics were defined using community surveys described in detail elsewhere [36]. Briefly, each village leader was interviewed in 2007 to determine the number of waterways and channels flowing into and out of each village, as well as the number and location of each reservoir. The head of each household was surveyed in 2007 and 2010 about agricultural practices, and this information was used to estimate the total area of agricultural cultivation in each village, and rice cultivation specifically. The research protocol was approved by the Sichuan Institutional Review Board and the University of California, Berkley, Committee for the Protection of Human subjects.

Percentage of land cover devoted to agriculture was determined using Landsat and IKONOS imagery according to classification schemes described previously [15]. A one kilometer buffer was rendered surrounding each village center in ArcGIS [37], and each cell within the buffer was characterized as either agricultural or non-agricultural (flowing water, barren, forest, built, other). The proportion of cells classified as agricultural was extracted for statistical analysis.

### Intermediate host sampling and genotyping

Between April 2008 and 2010, 833 *Oncomelania hupensis robertsoni* snails were hand-collected across the 29 sites from vegetation along small irrigation channels; snails were immediately preserved in 80% ethanol. Genomic DNA was extracted from the foot muscle or whole animals following our previous work [10]. We genotyped 11 polymorphic microsatellite loci, including 10 loci taken from Zhang et al. [44] and 1 loci (OH08) isolated for the current study from a microsatellite DNA library, produced by GENterprise (Mainz, Germany). Polymerase chain reactions (PCRs) were conducted in 10 μl reaction volume containing 10x ThermoPol reaction buffer, dNTPs (each 2.5 mM), 0.9 μl of each primer (each 10 μM), TMAC (tetramethylammonium chloride; 0.5 M), ddH_2_O, BSA (bovine serum albumin; 10 mg ml^-1^), 1 U Taq polymerase (New England Biolabs, Ipswich, Massachusetts, USA), and 1–50 ng DNA template. A fluorescent dye (Life Technologies Corporation, Carlsbad, California, USA and Metabion, Martinsried, Germany) was attached to the 5’ end of the forward primers. PCR cycling conditions were as follows: an initial denaturation step at 95°C for 5 min, followed by 35 amplification cycles (denaturation at 94°C for 40 s, annealing at the respective temperature for 40 s, and elongation at 72°C for 40 s), finalized by a terminal extension step at 72°C for 5 min. Prior to allele size determination, PCR products were pooled (3–4 loci per lot) depending on the expected size and the fluorescent dye used. The allele size determination was carried out on an ABI 3130xl Genetic Analyzer using the internal size standard GeneScan-500ROX (Life Technologies Corporation, Carlsbad, USA). Finally, the software GeneMarker version 1.90 (SoftGenetics LLC, State College, Pennsylvania, USA) was used for genotyping.

### Bayesian estimation of recent migration rates

Recent migrant rates (*m*_*ij*_, the expected proportion of migrants in site *j* that originated from site *i* within the most recent three generations) between populations were estimated using the Bayesian multilocus genotyping procedure implemented with BayesAss 3.03 [45]. Because this assignment technique identifies immigration occurring within the current and the previous two generations, assumptions regarding Hardy-Weinberg equilibrium, mutation, and effective population size are relaxed. However, the procedure works best with low levels of migration (i.e., <33%; [45, 46]) and well-structured populations (i.e., F_ST_ ≥ 0.05; [47]). To account for the latter aspect, we conducted preliminary F_ST_ analyses with our snail dataset using the software Microsatellite Analyser version 4.05 [48]. We found relatively high fixation index (F_ST_ = 0.15; p ≤ 0.001), justifying the subsequent use of BayesAss for estimating posterior probability distributions of individual migrant ancestries, population allele frequencies, and population inbreeding coefficients.

For the *Oncomelania* dataset, we conducted 10 Markov chain Monte Carlo (MCMC) runs, each with different seeds, 1x10^7^ iterations, discarding the first 1.5x10^5^ iterations as burn-in. Mixing parameters were adjusted for allele frequencies, inbreeding coefficients, and migration rates to reach an acceptance rate of 20–40% each [47]. To ensure that convergence was achieved, we also checked that non-migration rates and inbreeding coefficients for each site for each run were nearly identical, and determined the proportion of non-migration rates that were being drawn towards the upper and lower bounds [46]. In total, 28.3% of the non-migration rates estimated were within 10% of the lower bound (<0.73), and none of the non-migration rates fell within 10% of the upper bound (>0.90). We also examined log-probability plots created from each trace file for signs of stability and calculated Bayesian deviance for each run using Meirmans’ R script [46]. We considered the run exhibiting lowest deviance to be the optimal [47], and reran the analyses using the same seed and mixing parameters, increasing the number of iterations to 1x10^9^ and the burn-in to 2x10^7^. Resulting inter-village migration rates from these runs were used in subsequent analyses.

### Statistical analysis

Recent migration rates (logit transformed) of the *Oncomelania* snails were regressed against the three summary measures of all nine connectivity predictors in R using fixed effects models, which assume that each site has a constant baseline probability of attracting and retaining snail migrants, and random effects models, which allow for site-specific baselines [49, 50]. To-village immigration and from-village emigration were treated as two separate random effects relating to propensity of a village to attract migrant snails and retain native snails, respectively, controlling for the site’s geographical distance from other sites. The random intercepts generated for each village represent the respective attractivity and retentivity of each site [21]. In the random effects models, site attractivity and retentivity random effects were assumed to be independent and Gaussian.

Linear regression models were fitted to examine the ability of site-specific characteristics to predict the propensity (as measured by the random intercepts) of sites to attract migrant hosts and retain native hosts. Village-specific characteristics included: village elevation, number of streams into/out of the village, number of irrigation channels into/out of the village, village population, cultivated crop area, cultivated rice area, number of reservoirs per village, and land cover classification [36].

## Results

### Recent migration rates

Of snails sampled, 23.1% (n = 193) were identified as likely migrants within the most recent three generations by BayesAss assignment, having originated from a site other than the one from which the individual was sampled. The proportion of migrant individuals found at each study site ranged from 14.4% to 32.8%, with 20 of the 29 sites containing over 20% migrants. Fig 1 shows the proportion of the total snail population in each village that comprises recent migrant individuals. Site pairs (n = 87) between which inter-village migration rates (i.e., the proportion of migrants found in site *j* that originated from site *i* within the most recent three generations) above the average migration rate occurred were separated by Euclidean distances ranging from 0.3 to 44 km (mean: 11.8 km; median: 8.3 km).

Inter-village migration rates were low (mean: 0.87%, median: 0.62%, standard deviation: 1.50%); however, at eight sites, recent migrants from a single source comprised over 10% of the overall snail population. Three of these site pairs were within 1 km of each other, four pairs were within 2 km of each other, and one pair was separated by over 4 km. In the latter case, the destination site was ranked second of the 29 sites in its propensity to attract migrants as determined through ranking of random effect model intercepts.

### Influence of geographic distance and landscape connectivity on snail dispersal

Connectivity was significantly correlated (p<0.05) with migration rate for all nine connectivity models, with greater geographic distance (i.e., lower connectivity) between sites corresponding to a decrease in the odds of snail migration between the sites. Table 2 presents the relative odds of migration between villages given an increase of one interquartile range (IQR) in separation. Of the measures that summarize the distribution of geographic distances between a pair of sites, the minimum geographic distance explained the highest proportion of variance in migration rate across all models compared to the 25^th^ quartile and the median of the distribution. Based on the coefficient of determination, random effects models performed superior to fixed effects models in explaining migration rates.

**Table 2:** Odds ratios of migration for a one unit increase in IQR of the minimum geographic distance value for each of the connectivity models.

Environmental Weighting	Fixed Effects Model		Random Effects Model	
	OR	(95% CI)	OR	(95% CI)
Euclidean	0.820	(0.790, 0.849)	0.802	(0.721, 0.883)
Incline	0.797	(0.716, 0.879)	0.800	(0.719, 0.881)
Topography	0.766	(0.576, 0.955)	0.803	(0.722, 0.884)
Wetness	0.854	(0.787, 0.939)	0.811	(0.730, 0.892)
Land use	0.802	(0.721, 0.883)	0.812	(0.724, 0.899)
Watershed	0.812	(0.725, 0.899)	0.827	(0.746, 0.908)
Stream only	0.892	(0.807, 0.977)	0.891	(0.806, 0.976)
Streams and channels	0.827	(0.746, 0.908)	0.766	(0.576, 0.956)
Stream velocity	0.829	(0.762, 0.896)	0.890	(0.805, 0.975)

OR = odds ratio; CI = confidence interval

Because the majority of snail migration occurred within a few kilometers from the snail’s origin, connectivity models were compared on their ability to explain migration rate at a range of scales (Table 3). The large scale analysis, which included all site pairs, found that land use, topography, and incline models were the strongest predictors of inter-village migration rate on the basis of R^2^ values, with land use model performing the best of the three (Table 3). Land use models explained 14.8% of the variation in migration rate, while incline and Euclidean models explained 14.4% and 14.1% of the variation, respectively. Land use and watershed models performed the best even when considering migration between sites separated by a distance of less than 3 km.

**Table 3:** Relative coefficient of determinations from random-effects models for migration rate and geographic distance restricting sites to within specified distance, in meters. Highlighted boxes indicate superiority of fit, based on relative R^2^, where Euclidean model is used as the reference model. Higher numbers of R^2^ indicate superiority of fit.

Environmental Weighting	R^2^ Model/R^2^ Euclidean Model						
	1,000	2,000	3,000	5,000	8,000	10,000	All
Euclidean	1.00	1.00	1.00	1.00	1.00	1.00	1.00
Incline	0.63	2.13	1.22	1.12	1.07	1.05	1.02
Topography	0.70	1.22	1.07	1.03	1.01	1.01	1.01
Wetness	0.68	4.32	1.38	1.16	1.10	1.08	0.99
Land use	0.15	12.98	1.36	1.50	1.20	1.14	1.05
Watershed	1.18	4.62	1.52	1.31	1.18	0.08	1.00
Streams only	0.04	5.97	1.31	1.07	0.99	1.03	0.96
Stream velocity	0.08	5.70	1.30	1.03	0.96	1.01	0.95

### Influence of site characteristics on snail dispersal

The conditional and marginal coefficients of the random intercept models suggest that over 35% of the variance in migration rate can be attributed to village-level effects, while about 10% of the variance in migration rate can be attributed to landscape connectivity between villages. Intercepts from the random effects model for each site provide information on the site’s propensity to attract and to retain migrant snails. Sites with high attractivity, high retentivity, and low retentivity are shown in Fig 1.

Villages with larger area of land devoted to agricultural production were significantly more efficient at retaining migrants (p<0.05), though there was no indication that rice was more or less strongly associated with retentivity than other crop types (Table 4). However, controlling for irrigation decreased the association between agricultural fields and retentivity, due to high multicollinearity between irrigation channels and agricultural area. The number of irrigation channels leading into a village was significantly associated (p<0.05) with both high retention of snails and high attractivity of migrant snails.

**Table 4:** Associations between village characteristics and measures of site attractivity and retentivity of snails

Village Characteristic	Mean Difference^1^ (95% CI)	
	Retentivity	Attractivity
Population > 6 years old	17.25 (-4.47, 38.97)	-3.98 (-22.54, 14.58)
No. rivers entering village	0.36 (-0.07, 0.78)	0.10 (-0.27, 0.46)
No. irrigation channels entering village	0.91 (0.14, 1.45)*	0.53 (0.03, 1.02)*
No. rivers leaving village	0.28 (-0.13, 0.69)	0.03 (-0.31, 0.38)
No. irrigation channels leaving village	0.49 (-0.34, 1.32)	0.57 (-0.084, 1.23)
Proportion of irrigation channels lined with concrete	-2.13 (-19.19, 14.94)	-0.65 (-14.63, 13.32)
No. reservoirs	1.44 (-0.14, 3.03)	0.63 (-0.72, 1.99)
Area devoted to rice production (acres)	1.79 (-0.50, 4.08)	-0.13 (-2.50, 2.24)
Total agricultural area (acres)	9.86 (0.70, 19.01)*	0.90 (-4.77, 6.57)
Proportion of farmland devoted to rice cultivation	3.01 (-5.40, 11.42)	-0.61 (-8.93, 7.71)
Proportion of land cover cells within a 1 km buffer classified as agricultural^2^	0.47 (-10.88, 11.82)	3.07 (-6.12, 12.25)

CI = confidence interval

*indicates p<0.05

^1^ Reflects the mean difference in village characteristic units between sites ranking in the 75^th^ percentile for attractivity/retentivity and sites ranking in the 25^th^ percentile for attractivity/retentivity.

^2^ Obtained from remote satellite imagery. All other characteristics determined from village surveys conducted by Carlton and colleagues [36]

## Discussion

### Migration distances

This study involved sampling and genotyping of intermediate snail hosts in an area where re-emergence of schistosomiasis has occurred, and to our knowledge, is the first attempt at characterizing *O*. *hupensis robertsoni* migration through genetic assignment. Above average inter-village migration rates were observed between sites as far as 44 km apart, a distance that undoubtedly represents passive rather than active movement. *Oncomelania* snails can live over two years in some settings [51], and human or bird-mediated transport over this period is possible, as is rafting on vegetation in waterways. Van Leeuwen, et al. found that, in rare instances, snails ingested by birds may be transported long distances and excreted alive [52].

At the scale of migration distances observed, both active and passive snail transport may have important consequences for the transmission of schistosomiasis in areas that had previously attained disease control. The average lifespan of *S*. *japonicum*-infected snails has been estimated at 171 days, over which the examined snails shed an average of 673 cercariae [53]; thus a migrating infected snail has the capacity to contribute substantially to the parasite load in its destination site. The latency period following exposure to *S*. *japonicum* miracidia may provide ample time for snail hosts to migrate to an area previously free of schistosomiasis [54]. Moreover, migrating uninfected individuals may be more susceptible to parasite infection having not been exposed to *Schistosoma* spp. in their pre-migration environment [55].

We found that distances traveled by snails over a few generations in this study are comparable to the dispersal of other vectors, such as the malaria vector *Anopheles gambiae* [3], and the dengue vector A*edes aegypti* [56]. Given that both active and passive mosquito movement patterns are considered important to the spread of vector-borne disease [57–61] and can occur at scales similar to snail movement patterns, thorough investigation into the transmission impact of the snail dispersal reported in the present study is warranted. Eight of 536 village pairs comprised populations with recent migrant populations exceeding 10%, and each of these village pairs were within 5 km of each other, suggesting that unidirectional routes that are highly conducive to snail migration may exist between some communities.

### Landscape connectivity and site characteristics

Of the nine tested landscape connectivity models, land use best explained the observed variance in migration rates. Land use models consider agricultural lands and streams as conducive to movement, and barren land, built land, or standing water as prohibitive to movement. Previous studies have found that local snail dispersal occurs primarily along waterways [10] and that the hydrological connectivity of ponds and water pools after rainfall mediates the dispersal of snails within a suitable habitat [62]. Models presented here that included streams only and stream velocity were not consistently superior to Euclidean models at the full range of distances considered. However, the land use model, which accounts for stream features as part of the matrix determining connectivity, was consistently superior to Euclidean models, substantiating the importance of hydrological connections in determining snail distribution. Stream networks alone do not explain dispersal patterns; agricultural land use and the presence of barriers, such as barren or built land or standing water, are critical determinants.

Similarly, watershed models, which performed superior for sites separated by ≤3 km, consider the distance of each landscape cell to a stream cell, highlighting that snails require habitat at the interface of terrestrial and aquatic zones. The finding that minimum geographic distances between sites, as opposed to the 25^th^ percentile or median geographic distance, suggests that a single path of least resistance—one that lacks barriers and lies along suitable agricultural land and streams—between two sites can serve as an important corridor that facilitates migration between sites. The superiority of watershed models is also consistent with Liang et al., who found that, among all landscape fragmentation variables studied, wetlands showed the greatest correlation with genetic divergence of *Oncomelania* spp. [63]. This finding substantiates concerns over projects such as the Three Gorges Dam and the South-to-North water transfer project, which are expected to alter wetland patterns and could contribute to the expansion of suitable *Oncomelania* habitat [33, 63, 64].

Irrigation channels leading into a village were associated with increased likelihood that a snail would migrate to that village from a neighboring village and that a snail would be retained by that village. Area devoted to agricultural cultivation (as reported by the village leader) was also associated with an increase in the likelihood that a village would retain its native snail population, though the importance of area devoted to agricultural cultivation relative to irrigation channels decreased in multivariate analyses due to multi-collinearity. Since *Oncomelania* snails are amphibious organisms that require access to both water and littoral vegetation, villages with substantial irrigation for agriculture possess ideal habitats, and movements may be favored towards—and may terminate within—such areas that are conducive to snail survival. The number of irrigation channels leading away from a village had no association with the propensity of a village to attract or retain migrant snails.

### Limitations and future directions

While Bayesian assignment of genotypes in this study provides information on the putative origin and destination of migrating snails, the actual routes and means of dispersal between sites remain unknown. The estimation of migration rates was subject to the limitations of the Bayesian assignment methodology used [45], including that “unobserved” populations—those not captured in the survey and for which no reference allele frequencies are available—are not included as exchanging migrants, even if they are important sinks or sources. Additionally, more accurate estimates of migration have been shown to result from the analysis of as many as 20 loci, though estimates are believed to be accurate with as few as five loci genotyped in a population of at least 20 individuals if recent migration rates are low (i.e., ≤33%) [45]. The analysis presented here, following previous work, assumed migration rate was low (≤33%); yet for eight sites, the proportion of recent migrants was estimated to be over 32%, suggesting that true migration rates may have been higher than estimated. Such results may also indicate challenges in model convergence, just as where non-migration rates are observed near the lower bound [46]. However, the strength of population structure as measured by F_ST,_ as well as our inspection of the stability of the likelihood profile plots and prior values, increases confidence that model convergence was achieved in the present analysis.

Accounting for the effect of key landscape features on connectivity requires understanding snail preferences for, and movement behavior within, diverse landscape types, which have not been fully characterized for *Oncomelania* spp. [15]. Moreover, classifications of landscape types were themselves limited by the accuracy of classification procedures. Our model also excluded the effects of key time-variant determinants of intermediate host dispersal, including temperature and other seasonal effects, even as we acknowledge snail dispersal is known to be limited by cold microclimates [65]. However, recent work found that measures of geographic distance like those investigated here explained a larger proportion of the genetic divergence of *Oncomelania* spp. than did climatic and other variables [63]. At the same time, passive snail dispersal may also be mediated by human and animal movement, and these were not captured in our study. Construction of roads and railways such as the Greater Mekon Subregion (GMS) Chengdu-Kunming corridor and the GMS North-South Corridor through mountainous terrain will breach geographical barriers in the study region, potentially opening up new ranges into which snails may expand [55, 66].

Future work may consider the dispersal of infected snails as compared to uninfected snails, as studies have shown that parasitized snails move less than non-parasitized snails [67, 68]. Analyses that consider snail dispersal from upstream to downstream locations within irrigation channels would also be of interest considering the significant association found in this study between irrigation channels and snail retentivity and attractivity. Akullian et al. suggested that *O*. *hupensis robertsoni* snails are capable of moving both upstream and downstream, but found that snails dispersed further, on average, in the downstream direction [69]. Even slow moving flows in irrigation channels were observed to facilitate snail dispersal downstream, implying that hydrological models that explicitly consider the direction and magnitude of flows within waterways could be used in future analyses to improve upon the landscape models presented here, possibly leading to more targeted snail control interventions that account for connectivity.

The progress China has made towards elimination of schistosomiasis is threatened by an expansion of territory suitable for intermediate hosts under future climate and land use scenarios [33, 34, 64]. This study has important implications for snail control. Zhou et al. found that snail densities were lower in habitats that were highly fragmented, suggesting that habitat fragmentation may be a viable method for snail control centered around limiting snail dispersal [11]. New methods of snail control utilize Landsat satellite imagery to identify habitats suitable for snails [70], and then target interventions to snail-dense areas [71]. A combination of the analyses reported here with control programs based on landscape suitability could yield new control strategies that target where snails currently are, as well as where they may migrate, providing barriers to the establishment (or re-establishment) of host populations in new areas.

In conclusion, to the best of our knowledge, this is the first study aimed at quantifying migration of *O*. *hupensis robertsoni* using Bayesian analysis of multilocus genotype data. The analysis classified up to 20% of snails as recent migrants, suggesting there is potential for schistosomiasis to spread through the migration of intermediate hosts. Geographic distance was strongly correlated with snail migration, with models that include both water and land use features performing well for sites separated by as many as 67 km. Our results provide insight into the movement ecology of the intermediate host for *S*. *japonicum*, and may be useful for designing control measures that limit the expansion of the species range.

## Supporting Information

S1 Table Results of AMOVAs in *Oncomelania hupensis robersoni*. Scaling orders are populations (PP) and watersheds (SO); all values are given in % (*p*-values for all listed results of variation < 0.001).
(DOCX)

S2 Table Raw genetic information of sampled *Oncomelania huensis robertsoni* snails (XLSX)

S1 Fig Consensus tree based on Nei’s chord distances derived from allele frequencies at 11 microsatellite loci (NJ method of tree construction) of *Oncomelania hupensis robertsoni*. Bootstrap values (> 50) are indicated at each node. Colors refer to watersheds (for SO = 7, 6 groups).
(TIF)

S2 Fig Structure output for K = 30 (the lowest *K* value with the highest likelihood) grouped by populations of *Oncomelania hupensis robertsoni*. (TIF)
