# Supplementary material for: Genetic Evidence of Contemporary Dispersal of the Intermediate Snail Host of Schistosoma japonicum: Movement of an NTD Host Is Facilitated by Land Use and Landscape Connectivity
Source: PLoS Negl Trop Dis. 2016 Dec 15;10(12):e0005151. doi: 10.1371/journal.pntd.0005151 (PMC5157946; doi:10.1371/journal.pntd.0005151)
Supplement: S1 Table — Scaling orders are populations (PP) and watersheds (SO); all values are given in % (p-values for all listed results of variation < 0.001). (DOCX) [file pntd.0005151.s001.docx]

| **Source of variation** | **PP** | **SO=9**  (2 groups) | **SO=8**  (3 groups) | **SO=7**  (6 groups) | **SO=6**  (10 groups) | **SO=5**  (17 groups) | **SO=4**  (21 groups) |
| --- | --- | --- | --- | --- | --- | --- | --- |
| V_a_ (among groups of populations) |  | 2.95 | 3.56 | 4.14 | 8.25 | 7.36 | 9.62 |
| V_b_ (within groups of populations) |  | 14.10 | 13.68 | 12.17 | 8.11 | 8.61 | 6.22 |
| V_c_ (within populations) |  | 82.95 | 82.77 | 83.69 | 83.64 | 84.04 | 84.16 |
