# Supplementary figures and images for: Genetic Evidence of Contemporary Dispersal of the Intermediate Snail Host of Schistosoma japonicum: Movement of an NTD Host Is Facilitated by Land Use and Landscape Connectivity
Source: PLoS Negl Trop Dis. 2016 Dec 15;10(12):e0005151. doi: 10.1371/journal.pntd.0005151 (PMC5157946; doi:10.1371/journal.pntd.0005151)

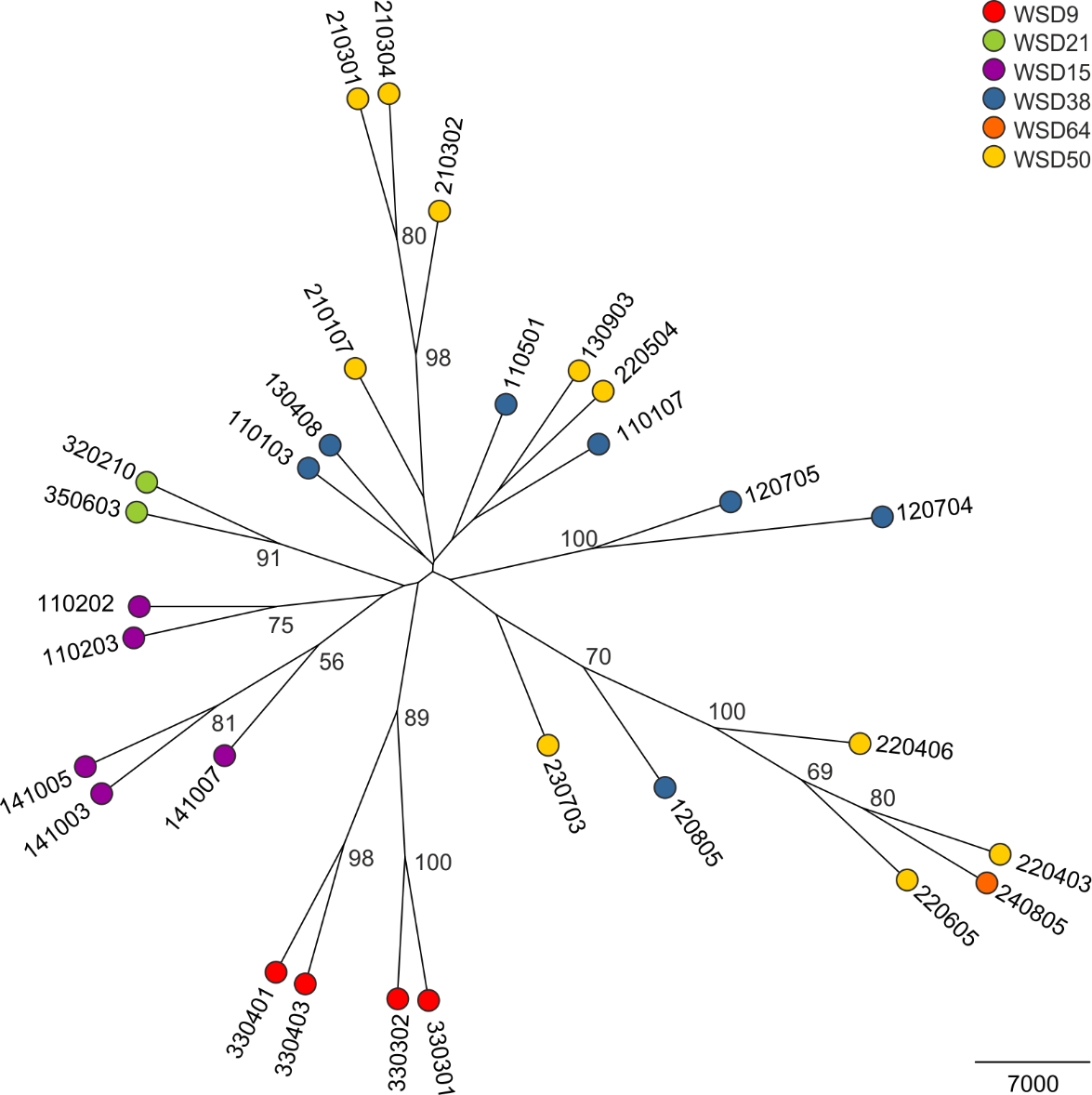

Supplement: S1 Fig — Bootstrap values (> 50) are indicated at each node. Colors refer to watersheds (for SO = 7, 6 groups). (TIF) [file pntd.0005151.s003.tif]

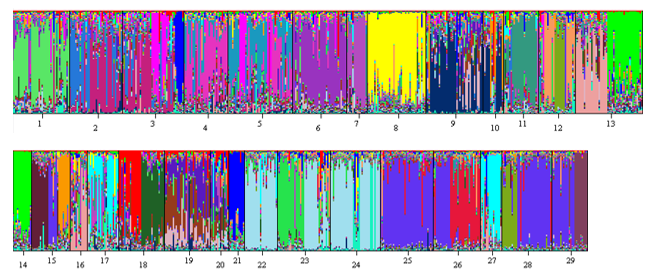

Supplement: S2 Fig — (TIF) [file pntd.0005151.s004.tif]
